# ‘Doing culture’ in contemporary south-eastern Australia: how Indigenous people are creating and maintaining strong cultural identities for improved health and wellbeing

**DOI:** 10.1186/s12889-024-19146-w

**Published:** 2024-06-26

**Authors:** Naomi Tootell, Janet McGaw, Uncle Herb Patten, Alasdair Vance

**Affiliations:** University of Melbourne, Melbourne, Australia

**Keywords:** Aboriginal and Torres Strait Islander, First Nations, Koori, Identity, *Yarns*, *Yarning*, Constructed Grounded Theory, *Country*, Culture, Stolen Generations

## Abstract

**Background:**

Indigenous people in Australia experience far poorer health than non-Indigenous Australians. A growing body of research suggests that Indigenous people who are strong in their cultural identity experience better health than those who are not. Yet little is known about how Indigenous people create and maintain strong cultural identities in the contemporary context. This paper explores how Indigenous people in south-eastern Australia create and maintain strong cultural identities to support their health and wellbeing.

**Methods:**

Data were collected from 44 Indigenous people living in the south-eastern Australian state of Victoria via *yarning*. *Yarning* is a cultural mode of conversation that privileges Indigenous ways of knowing, doing and being. *Yarning* participants were selected for their prominence within Victorian Indigenous health services and/or their prominence within the Victorian Indigenous community services sector more broadly. Due to the restrictions of COVID-19, *yarns* were conducted individually online via Zoom. Data were analysed employing constructivist grounded theory, which was the overarching qualitative research methodology.

**Results:**

All *yarning* participants considered maintaining a strong cultural identity as vital to maintaining their health and wellbeing. They did this via four main ways: knowing one’s *Mob* and knowing one’s *Country*; connecting with one’s own *Mob* and with one’s own *Country*; connecting with Community and *Country* more broadly; and connecting with the more creative and/or expressive elements of Culture. Importantly, these practices are listed in order of priority. Indigenous people who either do not know their *Mob* or *Country*, or for whom the connections with their own *Mob* and their own *Country* are weak, may therefore be most vulnerable. This includes Stolen Generations survivors, their descendants, and others impacted by historical and contemporary child removal practices.

**Conclusions:**

The *yarns* reveal some of the myriad practical ways that Indigenous people maintain a strong cultural identity in contemporary south-eastern Australia. While programs designed to foster connections to Community, *Country* and/or Culture may benefit all Indigenous participants, those most disconnected from their Ancestral roots may benefit most. Further research is required to determine how best to support Indigenous Victorians whose connections to their own *Mob* and their own *Country* are unable to be (re)built.

## Glossary

**Aboriginal and Torres Strait Islander** people are the **Indigenous** people of Australia. We have opted to mostly use the latter term in this paper. It is one of many terms used to collectively describe those with Ancestral links to the original inhabitants of the land and waters now known as Australia. None is uncontested as they tend to homogenise the many differences between groups [1]. We also provide a glossary here to assist an international audience with understanding some of the terms used throughout this paper. In particular, there are a number of English language words that take on specific meanings when used in the Australian Indigenous context. **Aunty** and **Uncle** are titles of respect for Indigenous women and men who are considered to carry significant knowledge and wisdom, also known as **Elders** ([2],p262). ***Country*** is a term used to refer to the lands, waters and skies to which a person is connected through Ancestral ties. It is inclusive of plants, animals, rocks, celestial bodies, *Ancestors, Totems, Stories* and *Spirits *of *Country. *To be ‘on-*Country*’ or ‘off-*Country*’ indicates whether someone is physically present on or distant from their own Ancestral lands [3]. ***Mob ***is an Aboriginal English term referring to one’s own kinship group. A person’s *Mob *is associated with a particular place or *Country* [4].*** Yarning*** describes a relational, circular mode of conversation that is inclusive of ‘deep listening’ [5]. ***Stories*** is an Aboriginal English term for knowledge, often shared orally [6]. ***Bush ***is a colloquial Australian term used to describe any area that is not developed, farmed or settled [7]. 

## Introduction

Aboriginal and Torres Strait Islander people – the Indigenous people of Australia – continue to experience far poorer health than their non-Indigenous counterparts [8–10]. While the gap in some areas appears to be narrowing, the most recent data indicate there is still a long way to go before Indigenous Australians achieve health parity with non-Indigenous Australians. For example, Indigenous Australians continue to experience disease burden at 2.3 times the rate of non-Indigenous Australians [11]. This is despite successive governments’ determination to ‘Close the Gap’ in Indigenous health over the past fifteen years [12].

The disproportionate burden of ill health carried by Indigenous people in Australia can be largely attributed to settler-colonialism – in both its historical and contemporary forms [13]. Complex Indigenous lifeworlds, created to ensure the health of Indigenous people, communities and *Country,* were severely disrupted by colonisation, and the settler-colonial lifeforms that displaced them have tended to be, at best, incapable of promoting Indigenous health, or at worst, actively destructive of Indigenous people and health [14]. This situation persists today. Indigenous people continue to be subjected to racism and discrimination, or to feel otherwise misunderstood or excluded within mainstream Western health settings [15]. And yet, despite the multiple atrocities and dislocations of colonialism, Indigenous people in Australia maintain a strong and vibrant contemporary presence – a testament to the resourcefulness, adaptability and resilience of Australia’s diverse Indigenous peoples, cultures and communities [16, 17].

A growing body of scientific literature suggests that identifying with and engaging in culture may have positive health benefits not only for Indigenous people living in Australia, but for minoritised groups living in Western societies more generally [18–23]. Studies have shown that possessing a strong cultural identity may increase self-esteem, foster resilience, promote pro-social coping styles and help reduce the risk of experiencing mental health issues [24–26]. Having a positive cultural identity may also help shield people against the harmful effects of racism and discrimination [27–29]. The findings of these studies are encouraging. They suggest that strengthening cultural identity may be one way of improving the health outcomes of Indigenous people. But they also invite a question: how might this be done?

The specific aspects of culture that promote and support the health and wellbeing of Indigenous people and communities have increasingly been identified [30]. While there is no consensus on categories, the focus tends to be on connection to various overlapping domains, including: connection to friends, family and the broader Indigenous community; connection to plants, animals, land, the environment and/or *Country*; and connection to the less tangible aspects of Indigenous culture including languages, values, beliefs, history, knowledge, creative or artistic practices and spirituality [30–33]. Some studies additionally note the importance of self-determination and leadership to the development of cultural identity – highlighting the broader social, economic and political structures and forces that allow for, and support, connections across the various domains [31]. Other studies highlight the significance of a person’s connection to their own mind, body and emotions in determining the cultural connections that are health-giving for them [33].

The existing literature provides some detail for how healthy connections across the various domains of culture are created and maintained [34–38]. There is also a small but growing body of research indicating what form or content a program designed to strengthen cultural identity might have [39–42]. This paper adds to this growing body of research. We ask how Indigenous people create and sustain strong cultural identities for themselves in the contemporary moment. Connection to Community, *Country* and Culture are generally deemed to be central [43], but what does this require or look like in practice? Are some connections more important than others? And how might those wishing to strengthen the Indigenous cultural identity of themselves or others (through the development of targeted programs, for instance) draw on this knowledge? These questions are particularly pertinent for Indigenous people living in the south-east of Australia, where colonisation has arguably had the most longstanding and deleterious impact [13]. But they are questions relevant to other places subject to cultural dispossession and dislocation. We draw on research *yarns* conducted with 44 Indigenous people from around the south-eastern Australian state of Victoria to answer these pressing questions.

## Background

Aboriginal and Torres Strait Islander peoples represent one of the oldest living cultures in the world [44]. Complex systems of values, beliefs, knowledges, social organisation and land management practices have ensured Aboriginal and Torres Strait Islander people’s health and survival in sometimes harsh and forbidding environments for more than 60,000 years [45]. Indigenous Australia is also incredibly diverse: at the time of European arrival on the continent, more than 250 distinct Indigenous languages were spoken, including approximately 800 dialects [46].

According to Indigenous ontology, *Ancestral Beings* created the world and everything in it, including humans, the law and language [46]. Each language group is responsible for caring for tracts of *Country* created by the *Ancestors* in accordance with the law of that *Country* [47]. In return, *Country* provides all that is needed [47]. *Country* includes the whole of the material world: land, waterways, sky, stars, all entities they support, and the dynamic forces that enliven them [48]. *Country* also includes the more-than-material world, including the law, language, *Stories*, *Ancestors* and *Spirits* [48]. Indigenous people keep *Country* and *Spirit* alive by caring for its ecosystems, passing on and re-telling its *Stories*, and participating in ceremonies, including rhythmic song and dance [49]. By doing so, Indigenous people fulfil their role in maintaining the relationship of everyone and everything to everything else according to *Ancestral* law [49].

The arrival of Europeans on the continent of Australia 235 years ago fundamentally disrupted Indigenous peoples’ ancient regimes of culture and care. Successive colonial government laws and policies sought to eliminate Indigenous people and cultures – firstly through denial, then through destruction and dispossession, and finally through ongoing assimilation into non-Indigenous society [50]. The south-eastern regions of the continent were worst affected. Of the almost 27 million people living in Australia today, approximately one million (3.8 per cent) identify as Indigenous [51]. In the south-eastern state of Victoria, there are approximately 70,000 Indigenous people, representing one per cent of Victoria’s total population of almost 7 million [51]. Around half of all Indigenous Victorians live in the regions. This contrasts sharply with non-Indigenous Victorians, 76 per cent of whom live in the state’s capital city [52]. Of the approximately 38 Indigenous languages endemic to Victoria, few are routinely spoken, although several are in the process of being reclaimed and revitalised [53]. Due to the dislocations of colonialism, many Indigenous people today are not living on their own Ancestral lands or *Country*. Indigenous Victorians’ Ancestral links to *Country* now traverse the continent. In fact, around 4,500 Indigenous Victorians (5.8 per cent) identify as having Torres Strait Islander heritage – a culturally distinct Indigenous group from an archipelago off the north-eastern coast of mainland Australia [51].

The dislocation and dispossession wrought by settler-colonialism continue to have profound and enduring effects. These effects are perhaps most evident in the gap between Indigenous and non-Indigenous people in terms of social and emotional wellbeing, or mental health [54]. In Victoria, Indigenous people are approximately three times more likely to report experiencing high or very high levels of psychological distress compared to other Victorians, and six times more likely to present to Emergency departments for alcohol or drug-related harm – and these differences are growing [55]. Arguably, two of the most severely impacted groups are survivors and descendants of the Stolen Generations – those who were forcibly removed from their birth families based on race between the 1890s and the 1970s. Survivors and descendants of the Stolen Generations constitute approximately 37 per cent of Victoria’s adult Indigenous population [56]. Both groups perform far worse than other Indigenous people across almost every social indicator, including health and mental health [57]. This phenomenon is known as ‘the gap within the gap’ in Indigenous health and wellbeing [56]. Meanwhile, Indigenous Victorian youth continue to be removed from their birth families at twenty times the rate of non-Indigenous children and young people, and this group also fares worse than other Indigenous people across a range of social indicators, including health and mental health [58]. There is clearly an urgent need to break these cycles of disadvantage. Western settler-colonial society has failed, and is continuing to fail, Indigenous people.

Despite colonisation’s dark history, Aboriginal and Torres Strait Islander peoples and cultures have shown remarkable resilience [59]. Even in the south-east, after years of repressive government policies and practices, Indigenous people maintain a strong and identifiable presence [16]. They have resisted, persisted, re-organised, survived and thrived [17]. Aboriginal Community-Controlled Organisations, established in the late 1960s and early 1970s, developed strengths-based, culturally safe support for Indigenous people [60]. They continue to provide culturally sensitive services and to advocate for improved conditions for Indigenous people today [60]. Cultural reclamation practices, begun in the 1980s and 1990s, are also revitalising Indigenous communities [61]. Strengths-based approaches to Indigenous health research, which draw on Indigenous ways of knowing, doing and being, are also growing and having a positive impact on health and wellbeing [62]. They are critical of the deficit discourses that compare Indigenous to non-Indigenous people in Australia solely through the negative [62]. The research project reported in this paper sits within a strengths-based paradigm. It draws on Indigenist research methods as well as Indigenous perspectives and experiences to explore the culture-strengthening activities and programs that may be most beneficial, and for whom, in the contemporary south-eastern Australian context.

## Methods

### Indigenous-governed research

The findings presented here form part of a broader research project that is Indigenous-conceived, Indigenous-governed, and Indigenous-led. This is important, as research on or about Indigenous people – both in Australia and globally – has historically been conceived and conducted by non-Indigenous people; its findings deployed as justifications or tools of subjugation and control of the Indigenous populations it presumes to want to ‘understand’ and ‘help’ [63, 64]. Indigenous-led research ensures that issues affecting Indigenous people are increasingly conceived, understood – and ‘solutions’ are also creatively imagined within – Indigenous knowledge systems [65, 66].

The project is overseen by a governing board of Indigenous Elders (of which author HP is a member). There is also a Project Advisory Group consisting of Indigenous health professionals. The project co-leads include an Indigenous child and adolescent psychiatrist (author AV) and a non-Indigenous architect (author JM). Others on the team include an Indigenous psychologist, one non-Indigenous and two Indigenous research assistants and a non-Indigenous sociologist (author NT). The project is located within, and supported by, the Indigenous health cultural unit of a major metropolitan hospital. The project also sits within a broader national research program led by an Indigenous academic and medical doctor. Further details about the study design are available in the research team’s earlier publications [9, 10, 59, 67–76]. The project was approved by the Royal Children’s Hospital Human Ethics Committee (2019.207/56941).

### Research design and methodology

The broader research project consists of two stages. The findings presented here emerged from stage one, which involved *yarning* with Indigenous community members from within the south-eastern Australian state of Victoria. *Yarns* were focused on the relationship between Indigenous culture and health, including mental health. The second stage of the project (which is beyond the scope of this paper) involved the development of an adjuvant cultural therapy program for young Indigenous people who are non-responsive to standard Western mental health management alone. The program was developed from the knowledge that emerged from stage one [72].

The overarching research approach was a hybrid of multi-perspectival, constructivist grounded theory and community-led participatory action research, using the Indigenist research method of *yarning.* The method has been described in detail in another paper [75]. *Yarning* is the most widely reported Indigenist method used for gathering information from research participants in health research [77]. Rather than following a pre-determined set of questions, *yarning* provides a culturally safe environment for participants to explore the topic in question. Indigenous ways of knowing, doing and being are privileged, and participants become active collaborators in the production of knowledge [78]. *Yarning* emphasises the role of participants as experts in their own lives and as co-creators of any theories developed from the research data [79].

### Participant recruitment

The governing board of Indigenous Elders identified potential participants for the research *yarns* based on their leadership and/or advocacy within the domain of Indigenous health services and/or the domain of Indigenous community services more broadly within the south-eastern Australian state of Victoria. In some cases, participants recommended others to *yarn* with, and these referrals were always followed up. The sampling method was therefore a mix of purposeful, snowball and convenience [80]. A total of 46 participants were recruited to the research. Two participants subsequently withdrew from the research, and their data was not included in the analysis.

### Data collection

The *yarns* were originally intended to be conducted in-person. However, due to the restrictions of COVID-19, the *yarns* were conducted individually via *Zoom*. Under normal circumstances, the online environment might introduce a level of formality and distance not conducive to *yarning*. However, the *yarns* were conducted while Victoria was experiencing some of the longest and most restrictive lockdowns to occur in the country. Participants were often relieved or even excited to be communicating with another person online. The researcher was also a member of Victoria’s Indigenous community and very often known personally to the participant. In this scenario, online face-to-face communication became a step closer to community, rather than a step away. The *yarning* sessions were invariably relaxed and informal, and researcher and participant were able to journey together in discussing the topics of culture, health and the relationship between the two. Questions put to the participants were along the following lines:

1. Can you tell me what culture means to you?
2. What sorts of cultural activities or practices do you do?
3. How does participating in culture affect your health and wellbeing?
4. How does culture help explain why you are unwell?
5. What sorts of things do you do to feel well?

The *yarns,* which ranged in length from 30 to 90 minutes, were video-recorded and transcribed prior to being emailed to participants for verification.

### Data analysis

Data was analysed via a constructivist grounded theory approach [81]. Transcriptions were independently reviewed and coded by a team of four researchers – two Indigenous and two non-Indigenous. Each of the ‘coders’ wrote memos and identified provisional themes before meeting in *Zoom* to discuss their findings. A total of four extended *Zoom* sessions were held where researchers discussed their coding and analysis with each other. Coders took it in turns to share what they had ‘discovered’ through each *yarn,* a different person leading the discussion each time. A process of deliberation followed. This multi-perspectival team-based approach to analysis ensured discursive-reflexivity to challenge biases in interpretation [82]. Individual researchers tended to focus on different aspects of the data, but there were also significant areas of overlap. Preliminary findings from the *yarns* were presented at a meeting of the research project’s governing Board of Indigenous Elders and the project’s Indigenous Advisory Group in December 2021. Feedback from the two groups was subsequently incorporated into the research findings. A broad overview of these findings has been published elsewhere [71]. The findings presented here focus on the knowledges and practices the *yarning* participants consider critical to maintaining their cultural identity and the impact this has on their sense of health and wellbeing.

## Results

### Participants’ demographic profile

The 44 *yarning* participants represent a diverse mix of genders, ages, locations and language groups. There were 4 women and 3 men aged between 20 and 40 years; 15 women and 5 men aged between 40 and 60 years; and 9 women and 8 men aged over 60 years. Slightly more than half of the participants were living in the state’s capital city, while the remainder were living in the regions. The split between those living in urban and regional areas was also spread relatively evenly across genders and age groups. More than 30 different language groups were represented by the 44 participants, including 18 different Indigenous Victorian groups. The other tribal affiliations were to groups connected to New South Wales, Tasmania and Queensland, including the Torres Strait Islands. Many participants identified Ancestral connections to more than one tribal group. Six were survivors of the Stolen Generations or their direct descendants.

All participants had worked in Indigenous-specific roles for most if not for the entirety of their careers. At the time of the *yarns*, more than half of participants were working in Aboriginal Community Controlled Organisations (ACCOs). Ten participants were employed within Indigenous units of mainstream organisations, and a further ten were either retired or working as independent consultants. More than half of participants were working in health or health related areas, including as psychologists, social workers, youth workers, Aboriginal health liaison officers, counsellors and therapists. Others were working in ACCOs that didn’t necessarily have a primary focus on health. Some were CEOs of those Indigenous organisations; others were Directors of Indigenous units within mainstream organisations. Some were academics and several would be widely recognised as Elders within the Victorian Indigenous community.

### Findings

*Yarning* participants were diverse in their articulations of what Culture means to them, how they maintain their own connections to Culture, and the impact of Culture on their health and wellbeing. Despite their many differences, however, there were some clear commonalities. Firstly, all participants considered maintaining a strong connection to Culture as critical to maintaining their health and wellbeing. This didn’t necessarily mean they saw Culture as a panacea. Most also saw a role for Western health interventions and treatments in supporting their health and wellbeing. But very few had experienced, or could envision experiencing, wellness for themselves without maintaining a strong connection to Culture.

Secondly, while *yarning* participants varied greatly in terms of the details of precisely how they maintain a strong connection to Culture for themselves, four common themes emerged. These were: Knowing your *Country* and Knowing your *Mob*; Connecting with one’s own *Country* and one’s own *Mob*; Connecting with *Country* and with Indigenous Community more broadly; and, Connecting with the more creative and/or expressive elements of Indigenous Culture. Importantly, almost all research participants revealed an order of priority in terms of which connections they considered most important to maintain. The order of priority was emphasised explicitly by some participants, but it was also evident in the extent to which each of the themes were discussed in the *yarns*. Each of the themes for maintaining a strong connection to Culture is detailed below, followed by a discussion of the impact of maintaining a strong Indigenous identity.

#### Knowing your *Country* and knowing your *Mob*

Almost all *yarning* participants stressed that the most important aspect of maintaining a strong cultural identity – for them – was knowing who their *Mob* is and, by extension, the specific tracts of *Country* they are connected to. Many participants pointed to the significance of this knowledge explicitly, but they also demonstrated the significance of this knowledge in the way they introduced themselves – by first acknowledging their *Mob* and their *Country*, and then acknowledging the *Mob* of the *Country* they were on.

*I’m a Wollemi man but I grew up on Gubbi Gubbi* Country *on the Sunshine coast and I now live in Melbourne on Wurundjeri* Country. (Participant 9, youth and community services worker)

*I think the most important thing is knowing your background, knowing your* Country*, knowing your people, knowing who you are related to. (Participant 27, retired community services worker and Elder)*

All 44 *yarning* participants knew which language groups they were connected to, including the six participants who were survivors of the Stolen Generations and their descendants. And all referred to their tribal connections in their introductions. Indeed, these things were almost always articulated first in the introduction – an indication of their cultural significance.

#### Connecting with one’s own *Country* and with one’s own *Mob*

The second most important aspect of maintaining a strong cultural identity emphasised by the *yarning* participants was regularly connecting with one’s own *Mob* and with one’s own *Country*. Many of the *yarning* participants indicated that living with their own *Mob* on their own *Country* – preferably not in a rural township, but out in the bush – would be ideal. In that case, the connections to both people and place can be regularly attended to, in person. Many of the *yarning* participants were living on their own Ancestral lands at the time of the *yarns*, albeit generally in a township, so this meant connecting to any *Mob* who lived close by was a regular part of everyday life. And even though they might be living in a built-up environment, the bush was never very far away.

*For me, what’s important is to always stay connected to family, knowing my family, knowing our history. Also going out on* Country*, fishing, going out bush walking … I love going for walks around Echuca on Yorta Yorta* Country*, walking along the Murray River because that’s home for me and I just find it extremely important to go back and recharge my battery as much as I can. (Participant 16, youth and community services worker)*

For those living off-*Country* (i.e. not on their own Ancestral lands), and especially for those living in the city, maintaining connections to their own *Mob* and to their own *Country* tended to require a more concentrated effort. Connecting with *Mob* was often done via the internet or over the phone. Participants spoke of regularly connecting with family members and extended kin to share news, seek guidance, offer advice, ask for permission (to use a particular language word in a particular context, for instance), or simply just to connect through *yarning*. Participants also spoke of returning home to *Country* – to their own Ancestral lands – as often as they were able. Depending on how far away they lived, visits might be weekly, or monthly. If a person’s *Country* was several states away, they might try to make the trip at least once per year.

*I have to go home once a year because my family would believe I’m too far from our* Mob*. It’s an agreement with our families that we all go home at least once a year, maybe not together, but we try and do a gathering every couple of years where we all go home at the same time, but where we can’t, we have to go home at least once a year. If you have children in your care, those children need to be on* Country *at least once a year. (Participant 20, healthcare case manager)*

All *yarning* participants, except for one, were very closely connected to their *Mob* and to their *Country*. Eighteen participants were living on their own Ancestral lands at the time of the *yarns*, although many had also lived off-*Country* at various points in time. Many others were currently living off their own Ancestral lands but had either grown up on-*Country,* or had lived on-*Country* at some point. Very few had never lived on their own Ancestral lands, and only one participant did not maintain strong connections to her own *Mob* and her own *Country*.

#### Connecting with Indigenous Community more broadly

While living in or visiting one’s own *Country*, and connecting with one’s own *Mob*, were generally considered paramount to maintaining a strong connection to Culture, all *yarning* participants also reflected on the importance of connecting with Indigenous people who weren’t necessarily from their own *Mob*. A number spoke of the (sometimes silent) acknowledgement Indigenous people offer one another when encountering each other in the street. Others noted the cultural obligation they feel they have bestowed upon them to welcome and include other Indigenous people into their network – especially those who may be disconnected from their own *Mob* or kin, or whose Elders may not hold the knowledges they have.

*We are lucky to have some wonderful, wonderful Elders in the Community that we call Aunty and Uncle because we love them, and they love us. They may not necessarily be our tribe or our blood, and people born off-Country or who live off-Country, they don’t [necessarily] have Elders of that standing that they know. I think we should be able to learn off others if they are willing to teach us, just for the survival of us all. (Participant 44, mental health worker)*

Participants spoke of connecting with people who were not from their own *Mob* or their own family by participating in local Indigenous sporting teams or clubs, and by attending local community events, such as protests, rallies, cultural celebrations, and sporting carnivals. Several participants spoke of the importance of local ACCOs, including co-ops and health centres, for fostering a sense of local Indigenous community. Participants considered this to be beneficial for those living on their own *Country*, as well as for those living off-*Country*, and as perhaps one of the best starting points for those who are disconnected from their *Mob* and Community altogether.

*One of my biggest things is make sure you go to Aboriginal organisations like VAHS [the Victorian Aboriginal Health Service], as there is always someone who knows someone, [and they’ll say], ‘Oh bub, I’ll introduce you.’ So, using those [Aboriginal] services, and role modelling as workers in there. It’s not just a place to get your medical, there is so much more to it. Even carnivals or sporting clubs which are specifically for our* Mob*, there is so much more in that. (Participant 3, social worker)*

#### Connecting with *Country* more broadly

Participants living off-*Country* who were unable to return home to *Country* regularly also spoke of the importance of connecting to *Country* more broadly – wherever they happened to be. They saw this as important for their own sense of identity and wellbeing. Connecting with *Country* might be as simple as going outside into the backyard and lighting a fire, or taking your shoes off and rubbing your feet into the earth. It might mean finding a nearby area, relatively undisturbed by colonialism, and spending time in it, walking around, taking notice of the birds and the animals and the waterways and the land formations. The air and the clouds and the wind and the seasons. More deeply, connecting with *Country* might be knowing and identifying specific plant or bird or animal species, learning how to catch or harvest these, perhaps even hunting and gathering, cooking and eating these.

*I might be a Yorta Yorta, Muthi Muthi river person over here, and a salt water Bunurong person over there, but if I can’t get there, I’ll go to the nearest water place that I can and take my shoes off, so it’s about those little things and reconnecting and* yarning *to your Ancestors. It’s different for each and every one of us. (Participant 17, community services worker and Elder)*

Several participants specifically expressed an expansive concept of *Country*, one that encompassed non-Indigenous animal and plant species, as well as landscapes that had been significantly transformed by colonialism. Many also referenced the numinous or *Ancestral Spirits* in relation to spending time on *Country*. *Ancestors* and *Spirits* were generally considered to protect, guide, welcome, or warn those who seek to pass through particular tracts of *Country*. For some, knowing which *Spirits* were there to guide and protect, and which must be appeased or avoided, and responding appropriately, was an important aspect of their Indigenous self-identity.

*I speak to the Ancestors, and my grandmother in particular, and ask them for support and guidance, and my uncles who have passed, [for them to] be able to lead me in the right direction. So, I do a lot of talking [laughs] … I thank the Ancestors for letting me travel on their* Country *and I ask the spirits to provide a safe path for me and my children, and I do that before I take any long journey regardless of where I’m going. (Participant 5, healthcare case manager)*

#### Connecting with the more creative and/or expressive elements of Indigenous Culture

Approximately two thirds of *yarning* participants also mentioned connecting with the more creative and/or expressive elements of Indigenous Culture as important for them in maintaining a strong cultural identity. Practices mentioned include: using Indigenous language names, speaking in language, singing, dancing, making music, painting, weaving, possum skin cloak making, wood carving, spear-making, fish-trap making, etching, drawing, and painting. In some cases, participants were engaging in the more creative and/or expressive aspects of Culture because they had been passed down to them from their Elders. More often, however, they had learnt the practices or were beginning to engage with them as adults, as they had not had those practices taught to them or passed down.

*I did my first dance last year when I was 60 and I danced with my* Mob*. That was powerful and made me feel really well and gives you a strength. It’s when you don‘t do it, or are denied as a person or a people, that makes everyone unwell. (Participant 7, healthcare worker and former CEO)*

Several *yarning* participants were active contributors to the revitalisation of cultural knowledges and practices, such as possum-skin cloak making. Some drew on historical records and artefacts to try and recreate the knowledge or practice, and they sometimes spoke of how they can feel the *Ancestors* and the *Spirits* speaking to them through the artefacts, or through *Country*, teaching and showing them the way. Others said they feel they can recreate traditional knowledges or practices even without any direct teaching, because the knowledge is ‘in their DNA’.

*You don’t have to know the weaving stitch to weave. You don’t have to know how to carve wood and what your symbols are, you can use a burning tool if you are at a workshop and just do your own little bit of artwork. It’s your Aboriginal art because you are an Aboriginal person. (Participant 38, educator and academic)*

#### The impact of maintaining a strong Indigenous cultural identity

All *yarning* participants indicated that maintaining a strong connection to Culture was vital to maintaining their overall health and wellbeing. When strongly connected to Culture, participants spoke of feeling calm, peaceful, centred, grounded, balanced, uplifted, energised, safe and strong. Participants spoke of connecting to *Country* or to Culture as a way of slowing down and blocking out the noise and the busyness of modern life, of rebalancing one’s mind, body and spirit, of becoming better connected with oneself. Some likened the practice of walking on or being in *Country*, and of making art, to the practice of meditation. Others spoke of the sounds and smells and tastes of *Country* – of drinking the water from the river, of eating game meat, of hearing the birds – and of how these things were healing for them.

*Honestly, I know if I need to be healed, it’s not about a doctor’s appointment, it’s about getting in the car, going up to Wallaga Lake and just standing on* Country *at Wallaga Lake. I can still hear the birds even if I’m not there. I can close my eyes and hear the noises of Wallaga Lake. So, going home to* Country*, I can put the worries away. I feel really light, everything lifts off you. It’s hard to describe, but time stands still, so if I need to be well, I can go up there and come back feeling 150 per cent. (Participant 41, healthcare worker and CEO)*

Almost all participants had worked primarily in Indigenous affairs, where they said they were regularly exposed to the vicarious traumas of colonisation, as well as the systemic racism and discrimination of Western processes and institutions. All participants spoke of the difficulties of having to constantly negotiate between two worlds – an Indigenous one and a settler-colonial one – and of how draining and demoralising this can be. They considered maintaining a strong cultural identity (by regularly connecting with *Country*, Community and/or Culture) as critical in allowing them to continuously heal from these experiences and regain the strength to carry on with their work.

*Working in trauma can be really confronting, and some days I need to just remove myself and I’ll go to the parklands at the back of the hospital. It was a place of peace for me, being out in nature. It felt spiritual to me. And I found out later that Wurundjeri mob used to gather there. I was blown away by that because I had a sense of attachment to that place. I wonder if that’s a spiritual connection. (Participant 6, healthcare case manager)*

Some participants who were survivors of the Stolen Generations or their descendants said they had grown up very disconnected from Community and Culture. They attributed most of their difficulties earlier in life to this disconnection, while crediting their improved state of mind and general sense of wellbeing to their more recent connection to Community and Culture.

*I wasn’t brought up in my traditional community as my mum wasn’t brought up in it either. So, I never learned my traditional cultural practices, and I was very disconnected through no fault of my own .... And that created that whole path of alcohol abuse, drug abuse, not having the confidence to get into relationships where I was safe. That continued until I was in my late 30s/early 40s when I actually started connecting to my* Mob* … And the way that I did that was by coming back to Gippsland where I already had a bit of support over the years and connected with the* Elders *and they classed me as part of the Community. I started doing contemporary type cultural practices and started dancing with the little ones and acting, and that was part of my healing as well. (Participant 13, support worker and mentor)*

Several participants worked specifically in programs that aimed to (re)connect Indigenous youth and adults to Culture. They spoke of how healing those programs and experiences can be for those involved. But all participants also spoke of how connecting to Community, *Country* and Culture is a constant work in progress. Being Indigenous might be something you are – ‘it’s in your DNA’ – but it’s also something you do.

*Many people think they have to magically know how to be Black. I’m still learning how to be Black [laughs]. It’s something we do. I think it’s a strength of Aboriginal people that we are lifelong learners. (Participant 40, artist, activist and academic)*

## Discussion

In pre-colonial society, Indigenous connection to Community, *Country* and Culture occurred in concert. People lived on *Country* in family groups, spoke in language, hunted, gathered and prepared traditional foods and healing medicines. They also engaged in cultural practices walking, *yarning*, enacting ceremonies, making and decorating material implements, and meeting up with other groups for negotiations, discussions and exchange [83]. People were inculcated into their societies’ particular ways of knowing, doing and being from birth – through immersion, by constantly learning and doing. What emerged through the *yarns* is that Indigenous people living in contemporary south-eastern Australia who maintain a strong cultural identity do so by participating in as many of these cultural activities as they can, as often as they can – or as often as they feel they need to, and as often as they are called upon by others to connect. Specifically, participants spoke of maintaining connections to family, Community, *Country* (including *Ancestors* and *Spirits*) and Culture as critical to maintaining a healthy mind, body and spirit. When these connections were well-maintained, participants said they felt strong, centred, healthy, peaceful and grounded. When participants sensed that their mind, body and/or spirit were becoming unwell, or if they were feeling overwhelmed, unbalanced or disconnected, then they would spend time reconnecting with *Mob*, Community, *Country* and/or Culture to try and heal themselves [84].

Findings from the research *yarns* align closely with previous research indicating that maintaining strong connections to family, Community, *Country* and Culture is key to maintaining a strong cultural identity [30–39]. What is novel in these findings is some of the detail and the emphasis, along with the specific focus on the contemporary south-eastern Australian context – a region where cultural practices have often been described as lost through colonisation [13]. In particular, the strong emphasis of participants on knowing one’s *Country* and knowing one’s *Mob*, and connecting with one’s own *Country* and one’s own *Mob* first and foremost, is significant. All *yarning* participants know their *Mob* and *Country*, and all except one regularly connect with their *Mob* and their *Country*. Participants considered these to be the most important things in helping them to stay strong, healthy and well. From this position of knowledge and strength, *yarning* participants living on-*Country* were able to welcome *Mob* from elsewhere into their Community and onto their *Country*. Meanwhile, those living off-*Country* were able to reach out and connect with the local Indigenous Community and *Country* in the place where they live, further strengthening their cultural identity. Some *yarning* participants had creative and/or expressive cultural knowledges and practices handed down to them or passed on when they were young. Others learnt them as adults. Some felt sufficiently strong in their cultural identity to begin to discover and experiment with these cultural practices themselves. Reclaiming, revitalising and/or engaging in these knowledges and practices further connected those *yarning* participants to Culture and helped strengthen their Indigenous sense of self. But, for all *yarning* participants, this process of creating and maintaining a strong cultural identity began with knowing who they are and where they come from.

All 44 *yarning* participants who were invited to participate in the research partly based on having a strong cultural identity also happened to know who they are and where they come from. This is unlikely to be a coincidence. Indigenous Victorians who are strong in their cultural identity are more likely to know who they are and where they come from, and to consider maintaining close connections with their own *Mob* and *Country* as the first, most important aspect of maintaining their health and wellbeing. Indigenous Victorians who don’t know who they are or where they come from, or for whom the connections to their own *Mob* and their own *Country* are weak, may therefore be most vulnerable. This likely impacts a large proportion of Indigenous Victorians, including many of those affected by historical and contemporary practices of child removal. Stolen Generations survivors, their descendants and others impacted by more contemporary child removal practices also experience worse health and wellbeing outcomes compared to other Indigenous Victorians [56, 85]. If having a strong cultural identity is as fundamental to health and wellbeing as the *yarning* participants insist, then the historical disconnection from Community, *Country* and Culture experienced by Stolen Generations survivors and others may help explain ‘the gap within the gap’ in health and other outcomes. What can be learnt from the *yarns* in terms of supporting those who have been disconnected from their Ancestral roots and/or birth families to foster or strengthen their connections to Community, *Country* and Culture, for the benefit of their health and welllbeing?

Findings from the *yarns* suggest that the first most important thing to do for those who don’t know who they are or where they come from, or for whom the connections to their own *Mob* and their own *Country* are weak, is to try and retrace or rebuild the connections to *Mob* and *Country*. One *yarning* participant strongly urged anyone in such a position to do precisely this. The *yarns* highlight why services such as Link-Up and the Koorie Family History Service, which have been developed to support Stolen Generations survivors and others to make those connections, are so vitally important – from a contemporary cultural perspective [86]. Such services should receive priority funding, as they provide arguably some of the most important services for some of the most vulnerable groups. Currently, however, family-finding services tend to be under-funded, and full records of child removals during the Stolen Generations era were often not kept, or can be difficult to access ([56],p16). There will therefore be some for whom the knowledge of who they are and where they come from is not recoverable, or for whom those connections are unable to be (re)built. Others may simply not be comfortable with this approach to strengthening cultural identity.

The advice from *yarning* participants for anyone in this position wanting to build a strong cultural identity for themselves is simply to begin to connect, and to keep on connecting, with *Country*, Culture and Community. The *yarns* provide many practical examples of how to do this. A person can begin to connect to the *Country* one is on, simply by spending time in *Country*, paying attention to the entities it supports and the dynamic forces that enliven it. One can also begin to learn or to teach oneself Indigenous creative and/or expressive cultural practices to strengthen their Indigenous sense of self. Finally, one might access services run by local ACCOs, join a local Indigenous sporting team or club, or attend local Indigenous community events, including protests, rallies, marches, celebrations and other events, as a way of connecting with the local Indigenous community. *Yarning* participants emphasised that maintaining a strong cultural identity by regularly connecting across the three domains of Community, *Country* and Culture is a continuous process of learning and doing – one that is possible no matter whose *Country* one is on, and regardless of a person’s level of prior knowledge or experience.

Those looking to strengthen their cultural identity may also access one of the many programs currently being run by Indigenous organisations (and some non-Indigenous organisations) across the state of Victoria. Some programs are targeted at specific ages, genders and/or language groups. Others are targeted at priority groups such as young people in out-of-home care, those in the prison system, and those with drug and alcohol misuse issues. Some have the specific intended purpose of improving health and wellbeing outcomes for Indigenous participants. All aim to strengthen cultural identity through fostering connections to Community, *Country* and/or Culture in some way. Examples include the separate weekly meetups for men, women, youth and Elders hosted by Mullum Mullum Indigenous Gathering Place [87]; the cultural camps and cultural activity days run by the Victorian Aboriginal Child Care Agency for Indigenous young people in foster care, residential care and kinship care [88]; the statewide Indigenous arts in prison and community program [89]; and the 16-week residential healing programs run for Indigenous adolescents at the Baroona Healing Centre [90].

Historically, there has been limited evaluation of culture-building activities and programs [39]. However, recent research suggests they can be effective in strengthening cultural identity and improving a range of social outcomes for participants, including in the areas of health and mental health [39, 40]. Findings from the *yarns* suggest that, for those who know who they are and where they come from, therapies that work to strengthen participants’ connection to their own *Mob* and their own *Country* might be most relevant or effective. Meanwhile, programs that foster connection to Community, *Country* and/or Culture more broadly are likely to be beneficial for all Indigenous participants, although those who are disconnected from their Ancestral roots may benefit most. We recommend that future evaluations are sensitive to any potential differences in the effectiveness of programs for these two groups.

In recent years there have been programs developed specifically to connect Stolen Generations survivors to Community, *Country* and Culture – and to one another [91]. Where evaluations exist, such programs have been deemed to be very effective [39, 92, 93]. However, more programs are needed, including programs that assist Stolen Generations survivors and their descendants to heal together ([56],p137). Healing for Stolen Generations survivors and their descendants is important as it is likely to lead to improved health and other social outcomes. However, it is also critical in stopping the cycle of Indigenous children being removed from birth families. Victoria has the highest rate of Indigenous children in out-of-home care of all states and territories in Australia [85]. Due to the impact of intergenerational trauma, Stolen Generations survivors and their descendants are more likely than other Indigenous people to have their own children removed from their care ([56],p112). Separating Indigenous children from their Indigenous kin is where disconnection from Community, *Country* and Culture often begins, when the aim really should be, according to *yarning* participants, to strengthen those connections whenever and wherever possible. Programs that assist families impacted by transgenerational trauma to stay together and heal together – in part by fostering connections to Community, *Country* and Culture – are vitally important to stem the rising tide of Indigenous children being separated from their families; to stop intergenerational trauma being passed on; to strengthen cultural identity; and ultimately, to improve social outcomes for Indigenous people, including in the areas of health and mental health.

The Elder-governed cultural therapy program developed in response to the *yarns* – stage two of this project – goes some way to addressing this programmatic gap. The program was designed specifically to strengthen the cultural identity of young Indigenous people accessing mental health services who were unresponsive to Western mental health treatments alone. However, many of the participants to date have been descendants of Stolen Generations survivors and/or living in out-of-home care placements, or are otherwise disconnected from *Country*, Culture and Community. The therapy focuses on connecting to the local *Country*, including *Ancestors* and *Spirits*, as well as connecting to some of the more creative or expressive elements of Culture, such as wood carving and etching. The therapy is inclusive of siblings, parents, grandparents and/or carers of the young people who are its primary focus, so it can be said to be fostering connections to *Mob* and/or family. Finally, the people leading and assisting in running the program are all Indigenous-identified members of the local community. In that sense, the program is fostering connections with Community as well. The program is still in the early stages, so the outcomes have not been fully analysed, but preliminary findings from *yarning* with Indigenous participants and cultural therapists suggest the effects are positive.

## Limitations and future directions

The research *yarns* included the perspectives and experiences of 44 Indigenous participants living in a state with around 70,000 Indigenous residents. Participants represented a broad range of ages, genders and language groups, as well as those living in both regional and urban areas. However, Indigenous Victorians impacted by historical and contemporary child removal practices were underrepresented in the sample. This is likely a consequence of the deliberate bias in our study design. Participants were invited to participate in the research partly based on the strength of their cultural identities. The fact that all participants knew who they were and where they came from, and all except for one were closely connected to their own *Mob* and their own *Country*, was an unintended consequence of this bias. As a result, the question of whether and how Indigenous Victorians who do not know who they are or where they come from, or for whom those connections are tenuous or weak, create and maintain strong cultural identities for themselves can only be answered tentatively here. Detailed future research with Indigenous people who grew up disconnected from Culture, but who managed to create strong cultural identities for themselves nonetheless, is necessary.

## Conclusion

The research *yarns* suggest that Indigenous Victorians who are strong in their cultural identity are more likely to know who they are and where they come from and to consider maintaining strong connections to their own *Mob* and their own *Country* as the most important aspect of maintaining their health and wellbeing. Indigenous Victorians who don’t know who they are or where they come from, or for whom the connections to their own *Mob* or their own *Country* are weak, may therefore be most vulnerable. These findings help explain why Indigenous Victorians impacted by historical and contemporary child removal practices – including the 37 per cent of Indigenous Victorian adults who are Stolen Generations survivors or their descendants [56] – fare considerably worse on average in terms of health and other outcomes compared to Indigenous Victorians who have not been affected by child removal practices. To be disconnected from one’s Ancestral roots is to have one’s cultural identity weakened, and this has implications for a person’s health and wellbeing.

The research *yarns* suggest that, while all Indigenous Victorians are likely to benefit from programs that aim to strengthen cultural identity, those most disconnected from their Ancestral roots may benefit most. In particular, the yarns highlight the vital significance of programs targeted specifically at Stolen Generations families and others who have been affected by historical and contemporary child removal practices. Such programs are important not only for strengthening cultural identity, but for healing past traumas, for preventing trauma from being passed on, and for reducing the current rate that Victorian Indigenous children are being removed from their families. Breaking the cycle that continues to separate Indigenous children from their families is necessary for achieving better health and other outcomes, because, as the research *yarns* suggest, cultural identity is critical to Indigenous health and wellbeing, and this is fostered first and foremost through family. While the *yarns* offer many practical examples of how those who know who they are and where they come from maintain a strong cultural identity by regularly connect to Community, *Country* and Culture, further research is required to determine how best to support Indigenous people and families who have been disconnected from their Ancestral roots to foster those connections.

## Data Availability

The datasets analysed in this study are drawn from Indigenous participants. As Indigenous datasets, they are under the control of the study’s Indigenous Victorian Traditional Custodian Elder’s Board and are not publicly available. Data are however available from the authors upon reasonable request and with the permission of the Victorian Traditional Custodian Elder’s Board.
